# Carbon Monoxide Reduces Neuropathic Pain and Spinal Microglial Activation by Inhibiting Nitric Oxide Synthesis in Mice

**DOI:** 10.1371/journal.pone.0043693

**Published:** 2012-08-22

**Authors:** Arnau Hervera, Sergi Leánez, Roger Negrete, Roberto Motterlini, Olga Pol

**Affiliations:** 1 Grup de Neurofarmacologia Molecular, Institut d’Investigació Biomèdica Sant Pau & Institut de Neurociències, Universitat Autònoma de Barcelona, Barcelona, Spain; 2 INSERM U955, Equipe 3, Faculty of Medicine, University Paris-Est, Creteil, France; University of Medicine & Dentistry of NJ - New Jersey Medical School, United States of America

## Abstract

**Background:**

Carbon monoxide (CO) synthesized by heme oxygenase 1 (HO-1) exerts antinociceptive effects during inflammation but its role during neuropathic pain remains unknown. Our objective is to investigate the exact contribution of CO derived from HO-1 in the modulation of neuropathic pain and the mechanisms implicated.

**Methodology/Principal Findings:**

We evaluated the antiallodynic and antihyperalgesic effects of CO following sciatic nerve injury in wild type (WT) or inducible nitric oxide synthase knockout (NOS2-KO) mice using two carbon monoxide-releasing molecules (CORM-2 and CORM-3) and an HO-1 inducer (cobalt protoporphyrin IX, CoPP) daily administered from days 10 to 20 after injury. The effects of CORM-2 and CoPP on the expression of HO-1, heme oxygenase 2 (HO-2), neuronal nitric oxide synthase (NOS1) and NOS2 as well as a microglial marker (CD11b/c) were also assessed at day 20 after surgery in WT and NOS2-KO mice. In WT mice, the main neuropathic pain symptoms induced by nerve injury were significantly reduced in a time-dependent manner by treatment with CO-RMs or CoPP. Both CORM-2 and CoPP treatments increased HO-1 expression in WT mice, but only CoPP stimulated HO-1 in NOS2-KO animals. The increased expression of HO-2 induced by nerve injury in WT, but not in NOS2-KO mice, remains unaltered by CORM-2 or CoPP treatments. In contrast, the over-expression of CD11b/c, NOS1 and NOS2 induced by nerve injury in WT, but not in NOS2-KO mice, were significantly decreased by both CORM-2 and CoPP treatments. These data indicate that CO alleviates neuropathic pain through the reduction of spinal microglial activation and NOS1/NOS2 over-expression.

**Conclusions/Significance:**

This study reports that an interaction between the CO and nitric oxide (NO) systems is taking place following sciatic nerve injury and reveals that increasing the exogenous (CO-RMs) or endogenous (CoPP) production of CO may represent a novel strategy for the treatment of neuropathic pain.

## Introduction

Neuropathic pain is a clinical manifestation characterized by the presence of allodynia and hyperalgesia and it is difficult to treat with the most potent analgesic compounds. The mechanisms contributing to this syndrome involve local inflammatory responses, changes in the plasticity of neuronal nociceptive pathways and activation of spinal microglia [1].

Nitric oxide (NO) synthesized either by neuronal (NOS1) or inducible (NOS2) nitric oxide synthase mediates numerous neuropathic pain symptoms via cGMP-PKG pathway activation [2]–[4]. Accordingly, the expression of both enzymes is up-regulated in the spinal cord and dorsal root ganglia of animals with neuropathic pain [5]–[7]. The hypersensitivity effects induced by nerve injury are significantly diminished or absent in NOS1 (NOS1-KO) and NOS2 (NOS2-KO) knockout animals [6], [8] or reversed by the administration of selective NOS, guanylate cyclase or PKG inhibitors [4], [5], [9]. Moreover, the intraperitoneal administration of a NO donor potentiates the mechanical and thermal hypersensitivity induced by neuropathic pain [10].

Carbon monoxide (CO) synthesized by heme oxygenases-1 (HO-1) or 2 (HO-2), is another gaseous neurotransmitter implicated in the modulation of nociceptive pathways. However, while HO-2 exerts a pronociceptive effect during neuropathic pain [11], HO-1 plays an important role in the modulation of acute inflammatory pain [12], [13]. Consequently, the expression of HO-2 increases after nerve injury and the mechanical and thermal hypersensitivity to pain induced by nerve injury has been shown to be markedly decreased in HO-2-KO mice [14], [15]. In contrast, the over-expression of HO-1 is associated with potent anti-inflammatory and antinociceptive effects during inflammatory pain [11], [12]. However, the exact contribution of CO synthesized by HO-1 in the modulation of the main symptoms of neuropathic pain induced by sciatic nerve injury remains unknown.

It is interesting to note that, similarly to NO, CO also activates the cGMP-PKG pathway. As a result, a cross-talk has been reported between these two gases in several *in vitro* and *in vivo* models. For instance, NOS2-derived NO as well as NO donors contribute to induction of HO-1 by cGMP-PKG-dependent pathway activation [16], indicating that NO is an important regulator of CO produced by HO-1 [17]–[19]. Studies *in vivo* also demonstrated that while the antihyperalgesic effects induced by CO depend on the integrity of the NOS pathways, the antinociceptive responses produced by NO are independent of CO [13]. Nevertheless, the possible interaction between the NO and CO systems during neuropathic pain has not been investigated.

Carbon monoxide-releasing molecules (CO-RMs) are a new class of chemical agents able to reproduce numerous biological effects of HO-1-derived CO [20]–[23]. Several authors has shown that CO-RMs and HO-1 induction using cobalt protoporphyrin IX (CoPP) exert potent anti-inflammatory effects in vivo [11], [24]. However, the possible antiallodynic and antihyperalgesic effects produced by these compounds during neuropathic pain have not been evaluated.

It is well known that microglia modulates several neuronal changes occurring during the development and maintenance of numerous chronic states, including neuropathic pain [1], [25]. Interestingly, the administration of inhibitors of microglial cells activation significantly reduces the behavioral symptoms of neuropathic pain [25]. Thus, we investigated the potential role of CO and HO-1-derived CO in the modulation of neuropathic pain as well as the possible mechanisms involved in this action. Specifically, in sciatic nerve-injured WT and NOS2-KO mice we evaluated: 1) the mechanical antiallodynic, thermal antihyperalgesic and thermal antiallodynic effects produced by the administration of two CO-RMs, tricarbonyldichlororuthrnium(II) dimer (CORM-2) and tricarbonylchloro (glycinate)ruthenium (II) (CORM-3) as well as a classical HO-1 inducer (CoPP) and 2) the effect of these treatments on the expression of HO and NOS isoforms as well as CD11b/c, a marker of microglia activation, in the dorsal root ganglia and spinal cord of these animals.

## Results

### Effect of CORM-2, CORM-3 and CoPP Treatments in WT and NOS2-KO Sciatic Nerve-injured Mice

Animals were intraperitoneally administered twice daily with CORM-2 (5 mg/kg), CORM-3 (5 mg/kg), CoPP (2.5 mg/kg) or vehicle for a period of 11 days after surgery. On days 1, 5 and 11 of treatment mice were sequentially assessed for mechanical allodynia, thermal hyperalgesia and thermal allodynia.

**Figure 1:**
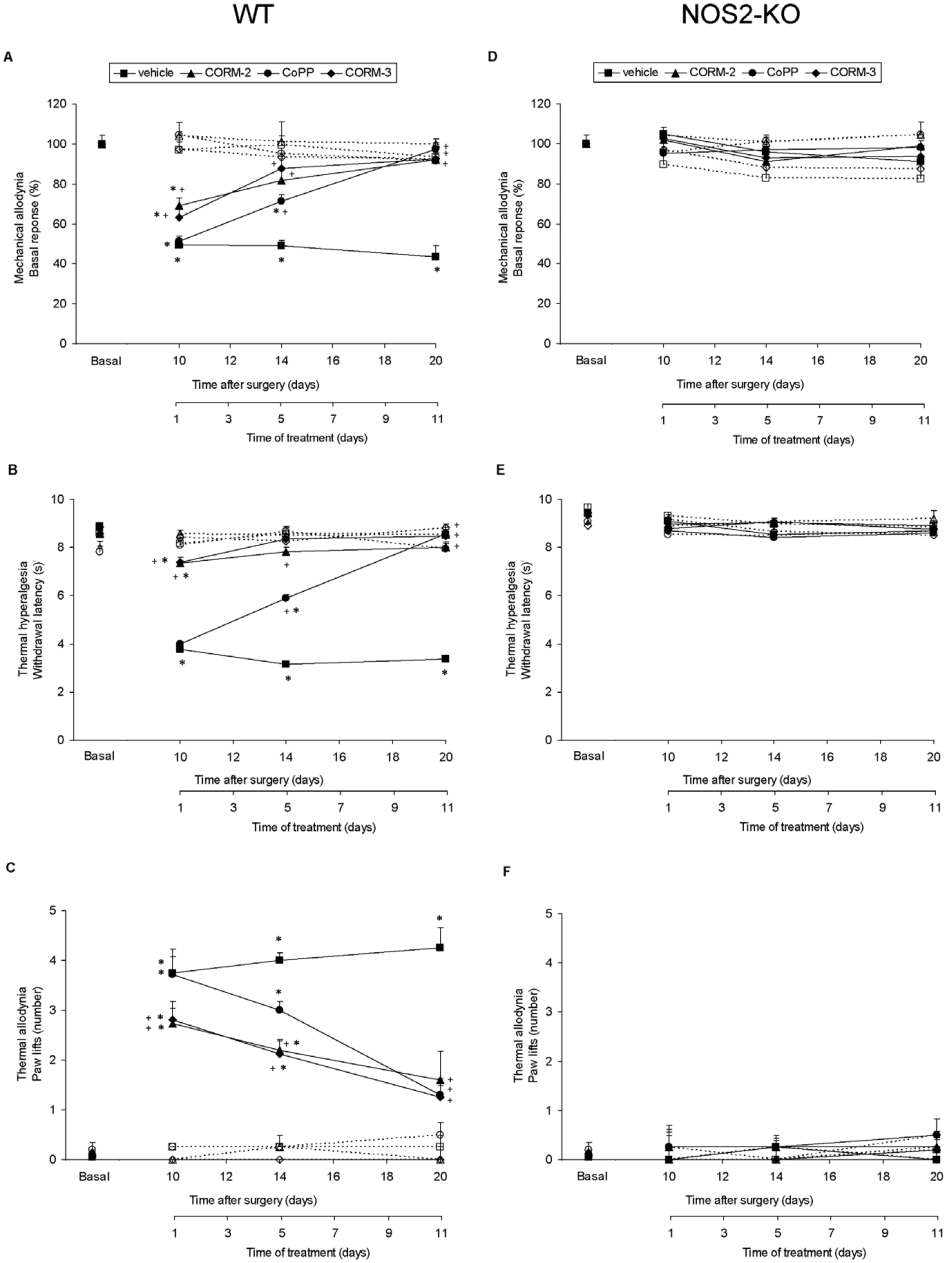
Effect of CORM-2, CORM-3 and CoPP on sciatic nerve-injured WT and NOS2-KO mice. The development of the mechanical allodynia (A and D), thermal hyperalgesia (B and E) and thermal allodynia (C and F) in sciatic nerve-injured (continuous lines) and sham-operated (discontinuous lines) WT or NOS2-KO mice treated during 11 consecutive days with vehicle, CORM-2, CORM-3 or CoPP at 10, 14 and 20 days after surgery is shown. For each genotype, test, day and drug evaluated, *indicates significant differences when compared vs. vehicle sham-operated group (p<0.001, one-way ANOVA followed by the Student Newman Keuls test) and +indicates significant differences when compared vs. vehicle nerve-injured group (p<0.001, one-way ANOVA followed by the Student Newman Keuls test). Results are shown as mean values ± SEM; n = 7 animals per experimental group.

Sciatic nerve injury led to a significant decrease of the threshold for evoking hind paw withdrawal to a mechanical stimulus in WT animals (Fig. 1A) which response was abolished in NOS2-KO mice (Fig. 1D). That is, mechanical allodynia was developed in vehicle treated WT mice exposed to sciatic nerve injury from days 10 to 20 after surgery when compared to sham-operated mice (p<0.001; one way ANOVA). This mechanical allodynia was significantly attenuated in nerve-injured WT mice repeatedly treated with CORM-2, CORM-3 or CoPP (Fig. 1A). The three-way ANOVA revealed a significant effect of the surgery, treatment and time (p<0.001) and a significant interaction between treatment and time (p<0.001), surgery and treatment (p<0.001), surgery and time (p<0.001) and between surgery, treatment and time (p<0.001). Indeed, mechanical allodynia was equally reduced on day 1 in CORM-2 and CORM-3, but not in CoPP, treated mice (p<0.001; one way ANOVA vs. vehicle nerve-injured treated mice), although the antiallodynic efficacy of all of them increased progressively on days 5 and 11 of treatment (p<0.001; one way ANOVA vs. vehicle nerve-injured treated mice). In sham-operated WT mice CORM-2, CORM-3 and CoPP treatments did not produce any effect as compared to sham-operated vehicle treated WT mice for the whole duration of the experiment.

The effects of CORM-2, CORM-3 or CoPP treatments in NOS2-KO mice exposed to sciatic nerve injury have been also evaluated. The three-way ANOVA did not reveal any significant effect of the surgery, treatment and time and non significant interaction between theme was demonstrated. Mechanical allodynia was not developed in NOS2-KO mice and the systemic administration of CORM-2, CORM-3 or CoPP did not alter the lack of mechanical allodynia observed in these nerve-injured animals (Fig. 1D). Sham operation did not produce any effect neither in CORM-2, CORM-3 or CoPP nor in vehicle treated NOS2-KO mice for the whole duration of the experiment.

Sciatic nerve injury led to a significant decrease of the threshold for evoking paw withdrawal to a thermal stimulus in WT mice from days 10 to 20 after surgery as compared to sham-operated mice (p<0.001; one way ANOVA). This thermal hyperalgesia was significantly attenuated in nerve-injured WT mice repeatedly treated with CORM-2, CORM-3 or CoPP (Fig. 1B). The three-way ANOVA revealed a significant effect of the surgery, treatment and time (p<0.001) and a significant interaction between treatment and time (p<0.001), surgery and treatment (p<0.001), surgery and time (p<0.001) as well as between surgery, treatment and time (p<0.001). Indeed, thermal hyperalgesia was completely blocked on day 1 in CORM-2 and CORM-3 treated WT mice (p<0.001; one way ANOVA vs. vehicle nerve-injured treated mice) and this level of efficacy was similarly maintained for both compounds on days 5 and 11 of treatment (p<0.001; one way ANOVA vs. vehicle nerve-injured treated mice). In contrast, thermal hyperalgesia was only significantly reduced by CoPP on day 5 (p<0.001; one way ANOVA vs. vehicle nerve-injured treated mice) and its antihyperalgesic efficacy increased progressively on day 11 of treatment (p<0.001; one way ANOVA vs. vehicle nerve-injured treated mice). In sham-operated WT mice CORM-2, CORM-3 and CoPP treatments did not produce any effect as compared to sham-operated vehicle treated WT mice for the whole duration of the experiment.

The effects of CORM-2, CORM-3 or CoPP treatments in NOS2-KO mice after sciatic nerve injury have been also evaluated. The three-way ANOVA did not reveal any significant effect of the surgery, treatment and time and non significant interaction between theme was demonstrated. Thermal hyperalgesia was not developed in NOS2-KO mice and the systemic administration of CO-RM’s or CoPP did not alter the absence of thermal hypersensitivy observed in these nerve-injured NOS2-KO animals (Fig. 1E). Sham operation did not produce any effect neither in CORM-2, CORM-3 or CoPP nor in vehicle treated NOS2-KO mice for the whole duration of the experiment.

Sciatic nerve injury increased the number of ipsilateral paw lifts during cold thermal stimulation in WT mice from days 10 to 20 after surgery as compared to sham-operated WT mice (p<0.001; one way ANOVA). This thermal allodynia was significantly attenuated in nerve-injured WT mice repeatedly treated with CORM-2, CORM-3 or CoPP (Fig. 1C). The three-way ANOVA revealed a significant effect of the surgery (p<0.001), treatment (p<0.001) and time (p<0.010) as well as a significant interaction between treatment and time (p<0.013), surgery and treatment (p<0.022), surgery and time (p<0.001) and the triple interaction between surgery, treatment and time (p<0.014). Indeed, although thermal allodynia was similarly reduced on days 1 and 5 in CORM-2 and CORM-3, but not in CoPP, treated WT mice (p<0.001; one way ANOVA vs. vehicle nerve-injured treated mice) the antiallodynic efficacy of all treatments increased progressively on day 11, where thermal allodynia was completely blocked by CORM-2, CORM-3 or CoPP treatments (p<0.001; one way ANOVA vs. vehicle nerve-injured treated mice). In sham-operated WT mice CORM-2, CORM-3 or CoPP treatments did not produce any effect as compared to sham-operated vehicle treated WT animals for the whole duration of the experiment.

**Figure 2:**
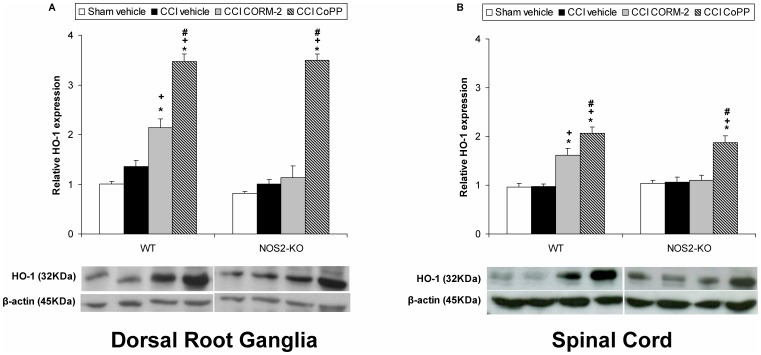
Effect of CORM-2 and CoPP on HO-1 protein expression from sciatic nerve-injured WT and NOS2-KO mice. The protein expression in the ipsilateral site of the dorsal root ganglia (A) and the lumbar section of the spinal cord (B) from sciatic nerve-injured (CCI) WT and NOS2-KO mice treated with vehicle, CORM-2 or CoPP at 20 days after surgery is represented. The expression of HO-1 in the dorsal root ganglia and spinal cord from sham-operated WT and NOS2-KO mice treated with vehicle has been also represented as controls (sham-vehicle). In both figures and genotypes, *indicates significant differences when compared vs. their respective sham-operated vehicle treated mice (*p<0.05, one-way ANOVA followed by the Student Newman Keuls test), +indicates significant differences when compared vs. their respective sciatic nerve-injured vehicle treated mice (+p<0.05, one-way ANOVA followed by the Student Newman Keuls test), #indicates significant differences when compared vs. their respective sciatic nerve-injured CORM-2 treated mice (#p<0.05, one-way ANOVA followed by the Student Newman Keuls test). Representative examples of western blots for HO-1 protein (32 kDa) in which β-actin (45 kDa) was used as a loading control are also shown. Data are expressed as mean values ± SEM; n = 5 samples per group.

The effects of CORM-2, CORM-3 or CoPP treatments in NOS2-KO mice after sciatic nerve injury have been also evaluated. The three-way ANOVA did not reveal any significant effect of the surgery, treatment and time and non significant interaction between theme was demonstrated. Thermal allodynia was not developed in NOS2-KO mice and the systemic administration of CORM-2, CORM-3 or CoPP did not alter the absence of thermal allodynia observed in these NOS2-KO nerve-injured animals (Fig. 1F). Sham operation did not produce any effect neither in CORM-2, CORM-3 or CoPP nor in vehicle treated NOS2-KO mice for the whole duration of the experiment.

In all experiments, CORM-2, CORM-3 or CoPP treatments did not have any significant effect in the contralateral paw of sciatic nerve-injured or sham-operated WT or NOS2-KO animals (data not shown).

### Effect of CORM-2 and CoPP on HO-1 and HO-2 Protein Expression in the Dorsal Root Ganglia and Spinal Cord from WT and NOS2-KO Sciatic Nerve-injured Mice

The protein levels of HO-1 in the dorsal root ganglia (A) and spinal cord (B) from sciatic nerve-injured WT or NOS2-KO mice treated with vehicle, CORM-2 or CoPP are shown in Fig. 2. For each tissue, the expression of HO-1 from sham-operated WT or NOS2-KO vehicle treated mice has been also shown. In both tissues, non-significant differences were found between genotypes as compared to the expression of HO-1 among them in vehicle sham-operated or vehicle sciatic nerve-injured mice treated with vehicle. However, while in sciatic nerve-injured WT mice the dorsal root ganglia and spinal cord expression of HO-1 was significantly increased by CORM-2 or CoPP treatments (p<0.001; one-way ANOVA vs. sham-operated and nerve-injured vehicle treated WT mice), in NOS2-KO mice the expression of this enzyme was only enhanced by CoPP (p<0.001; one-way ANOVA vs. to the other groups). In addition, the enhanced expression of HO-1 induced by CoPP in the dorsal root ganglia and spinal cord of sciatic nerve-injured WT mice is higher than those produced by CORM-2 treatment (p<0.001; one-way ANOVA followed by Student Newman Keuls test).

**Figure 3:**
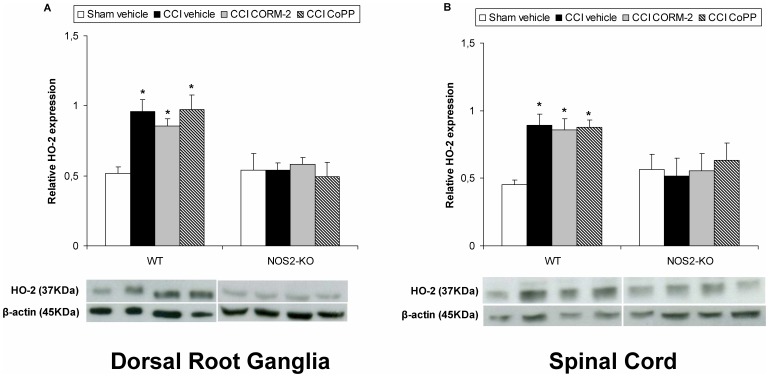
Effect of CORM-2 and CoPP on HO-2 protein expression from sciatic nerve-injured WT and NOS2-KO mice. The protein expression in the ipsilateral site of the dorsal root ganglia (A) and the lumbar section of the spinal cord (B) from sciatic nerve-injured (CCI) WT and NOS2-KO mice treated with vehicle, CORM-2 or CoPP at 20 days after surgery is represented. The expression of HO-2 in the dorsal root ganglia and spinal cord from sham-operated WT and NOS2-KO mice treated with vehicle has been also represented as controls (sham-vehicle). In both figures and genotypes, *indicates significant differences when compared vs. their respective sham-operated vehicle treated mice (*p<0.05, one-way ANOVA followed by the Student Newman Keuls test). Representative examples of western blots for HO-2 protein (37 kDa) in which β-actin (45 kDa) was used as a loading control are also shown. Data are expressed as mean values ± SEM; n = 5 samples per group.

The protein levels of HO-2 in the dorsal root ganglia (A) and spinal cord (B) from sciatic nerve-injured WT or NOS2-KO mice treated with vehicle, CORM-2 or CoPP are shown in Fig. 3. The expression of HO-2 from sham-operated WT or NOS2-KO mice treated with vehicle is also shown. Sciatic nerve injury significantly increased the dorsal root ganglia and spinal cord levels of HO-2 in vehicle, CORM-2 and CoPP treated WT mice (p<0.001; one-way ANOVA vs. sham-operated vehicle treated WT mice). In contrast, the dorsal root ganglia and spinal cord expression of HO-2 was not altered in vehicle, CORM-2 and CoPP treated nerve-injured NOS2-KO mice.

### Effect of CORM-2 and CoPP on CD11b/c Protein Expression in the Spinal Cord from WT and NOS2-KO Sciatic Nerve-injured Mice

**Figure 4:**
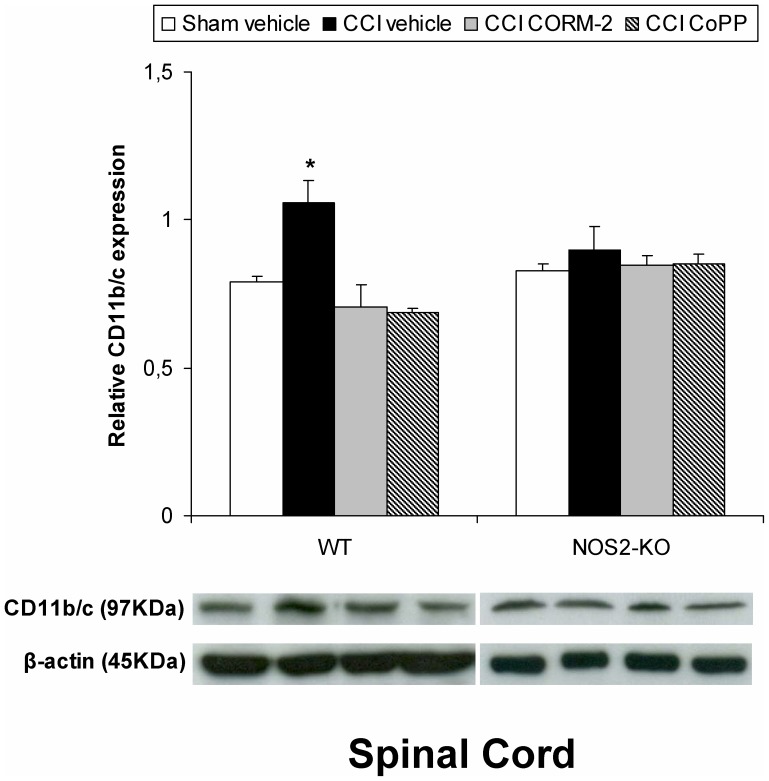
Effect of CORM-2 and CoPP on CD11b/c protein expression from sciatic nerve-injured WT and NOS2-KO mice. The protein expression in the ipsilateral site of the lumbar section of the spinal cord from sciatic nerve-injured (CCI) WT and NOS2-KO mice treated with vehicle, CORM-2 or CoPP at 20 days after surgery is represented. The expression of CD11b/c in the spinal cord from sham-operated WT and NOS2-KO mice treated with vehicle has been also represented as controls (sham-vehicle). In this figure *indicates significant differences when compared vs. sham-operated vehicle treated WT mice (*p<0.05, one-way ANOVA followed by the Student Newman Keuls test). Representative examples of western blots for CD11b/c protein (97 kDa) in which β-actin (45 kDa) was used as a loading control are also shown. Data are expressed as mean values ± SEM; n = 5 samples per group.

We next investigated whether the increased spinal cord expression of CD11b/c induced by nerve injury was altered by CORM-2 and CoPP treatments. The expression of CD11b/c from sham-operated WT or NOS2-KO vehicle treated mice is also evaluated (Fig. 4). Our results showed that the repeated treatment with CORM-2 and CoPP inhibited the increased expression of CD11b/c induced by sciatic nerve injury in WT mice (p<0.001; one-way ANOVA vs. sham-operated WT mice). Interestingly, sciatic nerve injury did not increase the protein levels of CD11b/c in NOS2-KO mice, which expression remains unaltered after CORM-2 or CoPP treatment.

### Effect of CORM-2 and CoPP on NOS1 and NOS2 Protein Expression in the Spinal Cord from WT and NOS2-KO Sciatic Nerve-injured Mice

**Figure 5:**
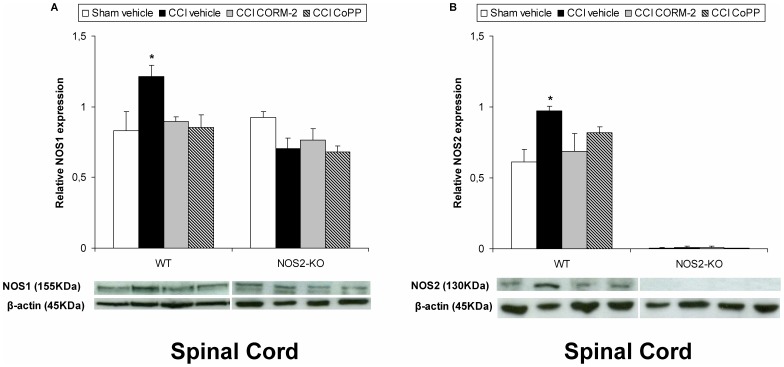
Effect of CORM-2 and CoPP on NOS1 and NOS2 protein expression from sciatic nerve-injured WT and NOS2-KO mice. The protein expression in the ipsilateral site of the lumbar section of the spinal cord of NOS1 (A) and NOS2 (B) from sciatic nerve-injured (CCI) WT and NOS2-KO mice treated with vehicle, CORM-2 or CoPP at 20 days after surgery is represented. The expression of NOS1 and NOS2 in the spinal cord from sham-operated WT and NOS2-KO mice treated with vehicle has been also represented as controls (sham-vehicle). In both figures and genotypes, *indicates significant differences when compared vs. their respective sham-operated vehicle treated mice (*p<0.05, one-way ANOVA followed by the Student Newman Keuls test). Representative examples of western blot for NOS1 (155 kDa) and NOS2 (130 kDa) proteins in which β-actin (45 kDa) was used as a loading control are also shown. Data are expressed as mean values ± SEM; n = 5 samples per group.

The protein levels of NOS1 (A) and NOS2 (B) in the spinal cord from sciatic nerve-injured WT or NOS2-KO mice treated with vehicle, CORM-2 or CoPP are shown in Fig. 5. The expression of NOS1 and NOS2 from sham-operated WT or NOS2-KO mice treated with vehicle has been also shown. Sciatic nerve injury significantly increased the protein levels of NOS1 and NOS2 in the spinal cord of WT mice (p<0.001; one-way ANOVA vs. sham-operated vehicle treated WT mice), which levels were significantly reduced by the repeated intraperitoneal administration of CORM-2 and CoPP. In contrast, sciatic nerve injury did not alter the spinal cord expression of NOS1 as well as of NOS2 in vehicle, CORM-2 or CoPP treated NOS2-KO animals, which protein levels were similar to those obtained in sham-operated NOS2-KO mice.

A summary of the results from the protein expression studies obtained in the ipsilateral site of the dorsal root ganglia and/or the lumbar section of the spinal cord from sciatic nerve-injured WT and NOS2-KO mice treated with vehicle, CORM-2 or CoPP is shown in Table 1.

**Table 1:** A summary of the results from protein expression studies obtained in the ipsilateral site of the dorsal root ganglia (DRG) and/or the lumbar section of the spinal cord (SC) from sciatic nerve-injured (CCI) WT and NOS2-KO mice treated with vehicle, CORM-2 or CoPP is shown.

		WT			NOS2-KO		
		CCI	CCI	CCI	CCI	CCI	CCI
Protein	Tissue	Vehicle	CORM-2	CoPP	vehicle	CORM-2	CoPP
HO-1	DRG	**→**	**↑**	**↑↑**	**→**	**→**	**↑↑**
	SC	**→**	**↑**	**↑↑**	**→**	**→**	**↑↑**
HO-2	DRG	**↑**	**↑**	**↑**	**→**	**→**	**→**
	SC	**↑**	**↑**	**↑**	**→**	**→**	**→**
CD11b/c	SC	**↑**	**→**	**→**	**→**	**→**	**→**
NOS1	SC	**↑**	**→**	**→**	**→**	**→**	**→**
NOS2	SC	**↑**	**→**	**→**	**→**	**→**	**→**

The arrows indicate: → unchanged, ↑ increased and ↑↑ more increased expression as compared to the expression obtained in their corresponding sham-operated animals treated with vehicle.

## Discussion

In the present study we demonstrated, for first time, that the repeated intraperitoneal administration of CORM-2, CORM-3 or CoPP significantly reduced the mechanical allodynia, thermal hyperalgesia and thermal allodynia induced by the chronic constriction of sciatic nerve in WT mice. Our results also indicate that these effects are mainly produced by CO synthesized by HO-1, through reduction of spinal microglial activation and NOS1/NOS2 over-expression.

It is well known that CO has potent antinociceptive and anti-inflammatory effects during inflammatory diseases [12], [24], [26]. In accordance, the results presented here further demonstrate that the intraperitoneal administration of CO by using CORM-2 and CORM-3 as well as the induction of endogenous CO by injection of the HO-1 inducer CoPP during 11 consecutive days significantly reduced the mechanical and thermal hypersensitivity induced by sciatic nerve injury in WT mice. Our results also revealed that, although at 11 days of treatment all compounds inhibited neuropathic pain with similar effectiveness, the antiallodynic and antihyperalgesic effects produced by CORM-2 and CORM-3 on days 1 and 5 of treatment were significantly higher than those produced by CoPP. These results could be related to the more direct release of CO by CO-RMs, in contrast to the HO-1 over-expression required by CoPP to synthesize CO. Our data also indicate that CO-RMs are more effective on the inhibition of thermal hyperalgesia than of mechanical and thermal allodynia induced by sciatic nerve injury.

In accordance to our previous studies [6], our findings showed that the principal manifestations of neuropathic pain induced by sciatic nerve injury were completely abolished in NOS2-KO mice. In addition, the lack of thermal and mechanical hypersensitivity observed in sciatic nerve-injured NOS2-KO mice remained unchanged after CO-RMs or CoPP treatments, similarly to that occurs in sham-operated WT or NOS2-KO treated animals.

As previously shown in other inflammatory models [27], [28], our results also demonstrated that the expression of HO-1 was significantly increased in the dorsal root ganglia and spinal cord of sciatic nerve-injured WT mice treated with CORM-2 or CoPP. As expected, the enhanced expression of HO-1 induced by CoPP (a HO-1 expression inducer) was higher than those produced by CORM-2 treatment. Interestingly, in sciatic nerve-injured NOS2-KO mice the expression of HO-1 was only increased after CoPP treatment. These results indicate that CORM-2 requires the presence of NOS2 to enhance the expression of HO-1, revealing that NO plays a key role in the positive feedback regulation of HO-1 over-expression during neuropathic pain. In accordance to our results other studies have been also demonstrated that the antihyperalgesic effects induced by CO in acute pain depend on the integrity of NO pathway [13].

In contrast to HO-1-derived CO, CO synthesized by HO-2 contributes to the progression of neuropathic pain. Thus, the lack of HO-2 appears to prevent the mechanical and thermal hypersensitivity to pain induced by nerve injury and the expression of this enzyme increases during neuropathic pain [14], [15], [29]. Our data support these findings and also demonstrate that the increased expression of this isoenzyme in the dorsal root ganglia and spinal cord of sciatic nerve-injured WT mice is not altered by CORM-2 or CoPP treatments, indicating that the antiallodynic and antihyperalgesic effects produced by the chronic administration of both compounds are not produced by the inhibition of the enhanced peripheral or central expression of HO-2 induced by nerve injury. In addition, the fact that the expression of HO-2 did not increase in the spinal cord and dorsal root ganglia of sciatic nerve-injured NOS2-KO mice, provide evidence that the up-regulation of HO-2 induced by nerve injury required the presence of NOS2, further supporting the relevant interactions between NOS/NO and HO/CO pathways previously demonstrated in other *in vivo* and *in vitro* models [11].

The molecular mechanism implicated in the inhibitory effects produced by CO after neuropathic pain is currently unknown. It has been reported that nerve injury promotes the activation of spinal glial cells, and that this activated glia may contribute to the initiation and maintenance of neuropathic pain [30]. Indeed, the administration of inhibitors of microglial cells activation significantly reduced the behavioral symptoms of neuropathic pain [25]. Several studies also demonstrated the presence of HO-1 in glial cells [31], but the possible effect of CO liberated by CORM-2 or synthesized by HO-1 on the modulation of activated microglia induced by nerve injury is not yet well established. Thus, in order to evaluate if this gas could reduce microglial activation and to establish the role played by NO, synthesized by NOS1 and NOS2, in this process we evaluated the expression of CD11b/c (as a measure of microglial activation), as well as of NOS1 and NOS2 in the spinal cord of sciatic nerve-injured WT mice treated with CORM-2 or CoPP. It is interesting to note that CORM-2 and CoPP treatments reduced the spinal microglial activation as well as the enhanced NOS1 and NOS2 expression induced by sciatic nerve injury in WT mice. Thus, the alleviation of the behavioral manifestations of neuropathic pain in CO-RMs or CoPP-treated WT animals could be due to the inhibition of inflammatory responses that are linked to the microglia activation in the spinal cord. In contrast to WT mice, the expression of CD11b/c and NOS1 remains unaltered after nerve injury in NOS2-KO mice and neither CORM-2 nor CoPP treatment had any effect in these animals. These results support the hypothesis that the activation of NOS/NO pathway promotes the activation of microglia and contributes to the behavioral pain responses evoked by nerve injury, as previously demonstrated by the lack or reduced mechanical and thermal hypersensitivity induced by nerve injury in NOS2-KO mice [6], [8].

Recent studies indicate that CORM-2, but not CORM-3, is also an antagonist of P2X4 receptors [32] and it is well known that the up-regulation of these receptors in microglia is an important process in producing neuropathic pain [33]. However, the similar behavioral inhibitory effects produced by CORM-2 and CORM-3 in the present study indicate that P2X4 receptors are not the main molecular targets for the antinociceptive effects produced by CORM-2 under neuropathic pain conditions. The modulation of neuropathic pain by the HO-1/CO pathway after sciatic nerve injury could be explained by the inhibition of excessive NO generated by the increased NOS1 expression from activated neurons, which plays an important role in the maintenance of neuropathic pain trough microglial activation. The activated microglia promotes the consolidation and progression of neuropathic pain by the up-regulation of several inflammatory pathways including the NOS2/NO pathway, among others. Thus, the activation of the HO-1/CO pathway on microglial cells would control and limit the spreading of this neuroinflammatory process by regulating the enhanced expression of NOS2. In addition, CO located in neurons could also participates in the modulation of neuropathic pain by decreasing the production of NOS1 which would restrict the activation of microglia and attenuates the development of neuropathic pain.

This study reports for first time that an interaction between the CO and NO systems is taking place following sciatic nerve injury. Our data also reveal that exogenous delivery of CO using CO-releasing molecules or increasing the endogenous CO production with cobalt protoporphyrin IX may represent a novel stratagem in the management of neuropathic pain.

## Materials and Methods

### Ethics Statement

Animal procedures were conducted in accordance with the guidelines of the European Communities, Directive 86/609/EEC regulating animal research and approved by the local ethical committee of our Institution (Comissió d’Etica en l’Experimentació Animal i Humana de la Universitat Autònoma de Barcelona, #6266). All efforts were made to minimize animal suffering, and to reduce the number of animals used.

### Animals

In vivo experiments were performed in male NOS2-KO mice (C57BL/6J background) purchased from Jackson Laboratories (Bar Harbor, ME, USA) and in WT mice with the same genetic background (C57BL/6J) acquired from Harlan Laboratories (Barcelona, Spain). All mice weighing 21 to 25 g were housed under 12-h/12-h light/dark conditions in a room with controlled temperature (22°C) and humidity (66%). Animals had free access to food and water and were used after a minimum of 6 days acclimatization to the housing conditions. All experiments were conducted between 9:00 AM and 5:00 PM.

### Induction of Neuropathic Pain

Neuropathic pain was induced by chronic constriction of the sciatic nerve [6]. Briefly, sciatic nerve ligation was performed under isoflurane anesthesia (3% induction, 2% maintenance). The biceps femoris and the gluteus superficialis were separated by blunt dissection, and the right sciatic nerve was exposed. The injury was produced by tying three ligatures around the sciatic nerve as described by Bennett and Xie [34]. The ligatures (4/0 silk) were tied loosely around the nerve with 1 mm spacing, until they elicited a brief twitch in the respective hindlimb, which prevented over-tightening of the ligations, taking care to preserve epineural circulation. Sham-operated mice that underwent exposure of the right sciatic nerve without ligature were used as controls.

The development of mechanical and thermal allodynia as well as thermal hyperalgesia was evaluated by using the von Frey filaments, cold plate and plantar tests, respectively. All animals were tested in each paradigm before surgery and at 10, 14 and 20 days after sciatic nerve injury.

### Nociceptive Behavioral Tests

Mechanical allodynia was quantified by measuring the hind paw withdrawal response to von Frey filament stimulation. In brief, animals were placed in a Plexiglas® box (20 cm high, 9 cm diameter) with a wire grid bottom through which the von Frey filaments (North Coast Medical, Inc., San Jose, CA, USA) bending force range from 0.008 to 3.5 g, were applied by using a modified version of the up–down paradigm, as previously reported by Chaplan et al [35]. The filament of 0.4 g was used first and the 3.5 g filament was used as a cut-off. Then, the strength of the next filament was decreased or increased according to the response. The threshold of response was calculated from the sequence of filament strength used during the up–down procedure by using an Excel program (Microsoft Iberia SRL, Barcelona, Spain) that includes curve fitting of the data. Clear paw withdrawal, shaking or licking of the paw were considered nociceptive-like responses. Both ipsilateral and contralateral hind paws were tested. Animals were allowed to habituate for 1 h before testing in order to allow an appropriate behavioral immobility.

Thermal hyperalgesia was assessed as previously reported by Hargreaves et al [36]. Paw withdrawal latency in response to radiant heat was measured using the plantar test apparatus (Ugo Basile, Italy). Briefly, mice were placed in Plexiglas boxes (20 cm high ×9 cm diameter) positioned on a glass surface. The heat source was positioned under the plantar surface of the hind paw and activated with a light beam intensity, chosen in preliminary studies to give baseline latencies from 8 to 9 s in control mice. A cut-off time of 12s was used to prevent tissue damage in absence of response. The mean paw withdrawal latencies from the ipsilateral and contralateral hind paws were determined from the average of 3 separate trials, taken at 5 min intervals to prevent thermal sensitization and behavioral disturbances. Animals were habituated to the environment for 1 h before the experiment to become quiet and to allow testing.

Thermal allodynia to cold stimulus was assessed by using the hot/cold-plate analgesia meter (Ugo Basile, Italy), previously described by Bennett and Xie [34]. The number of elevations of each hind paw was recorded in the mice exposed to the cold plate (4±0.5°C) for 5 minutes.

### Western Blot Analysis

Sham-operated and sciatic nerve-injured mice were sacrificed at 20 days after surgery by cervical dislocation. Tissues from the ipsilateral lumbar section of spinal cord and dorsal root ganglia (L3 to L5) were removed immediately after sacrifice, frozen in liquid nitrogen and stored at −80°C until assay. Samples from the spinal cord and dorsal root ganglia from three to five animals were pooled into one experimental sample to obtain enough protein levels for performing the western blot analysis. The HO-1, HO-2, CD11b/c, NOS1 and NOS2 protein levels were analyzed by Western blot. Tissues were homogenized in ice-cold lysis buffer (50 mM Tris·Base, 150 nM NaCl, 1% NP-40, 2 mM EDTA, 1 mM phenylmethylsulfonyl fluoride, 0.5 Triton X-100, 0.1% SDS, 1 mM Na_3_VO_4_, 25 mM NaF, 0.5% protease inhibitor cocktail, 1% phosphatase inhibitor cocktail). All reagents were purchased at Sigma (St. Louis, MO, USA) with the exception of NP-40 from Calbiochem. The crude homogenate was solubilised 1 hour at 4°C, sonicated for 10 seconds and centrifugated at 4°C for 15 min at 700×g. The supernatants (50 or 100 µg of total protein) were mixed with 4× laemmli loading buffer and then loaded onto 4% stacking/10% separating SDS-polyacrylamide gels. The proteins were electrophoretically transferred onto PVDF membrane for 120 minutes for HO-1, HO-2 and CD11b/c or over night for NOS1 and NOS2 detection, blocked with PBST +5% nonfat dry milk, and subsequently incubated overnight at 4°C with a polyclonal rabbit anti-HO-1 (1∶300, Stressgen, Ann Arbor, MI), a polyclonal rabbit anti-HO-2 (1∶1000, Stressgen, Ann Arbor, MI), a polyclonal rabbit anti-CD11b/c (1∶300, Novus Biologicals) antibody against the type 3 complement receptor to detect activated microglial cells [25], a polyclonal rabbit anti-NOS1 antibody (1∶100, BD Transduction Laboratories, San Diego, CA, USA) or a polyclonal rabbit anti-NOS2 antibody (1∶200, Chemicon, Millipore). The proteins were detected by a horseradish peroxidase-conjugated anti-rabbit secondary antibody (GE Healthcare, Little Chalfont, Buckinghamshire, UK) and visualized by chemiluminescence reagents provided with the ECL kit (Amersham Pharmacia Biotech, Piscataway, NJ, USA) and exposure onto hyperfilm (GE, Healthcare). The intensity of blots was quantified by densitometry. The membranes were stripped and reproved with a monoclonal rabbit anti-β-actin antibody (1∶10.000, Sigma, St. Louis, MO, USA) used as a loading control.

### Experimental Protocol

In a first set of experiments we assessed the expression of neuropathic pain by using the mouse model of the chronic constriction of sciatic nerve previously used by us [6]. After the habituation period, baseline responses were established in the following sequence: von Frey filaments, plantar and cold plate tests. After that neuropathic pain was induced, and WT or NOS2-KO animals were again tested in each paradigm at days 10, 14 and 20 after surgery. Sham-operated mice were used as controls.

Sciatic nerve-injured or sham-operated WT or NOS2-KO animals received the intraperitoneal administration of two CO-RMs (CORM-2 or CORM-3, at 5 mg/kg of body weight twice a day) [21], [27], an HO-1 inducer (CoPP, at 2.5 mg/kg of body weight twice a day) [37] or vehicle, from days 10 to 20 after surgery.

In other set of experiments, taking into account the analogous effects produced by CORM-2 and CORM-3 on the inhibition of the allodynia and hyperalgesia induced by sciatic nerve injury and in order to minimize the number of animals used, the protein levels of HO-1, HO-2, CD11b/c, NOS1 and NOS2 in the ipsilateral site of the spinal cord and/or dorsal root ganglia from sciatic nerve-injured WT and NOS2-KO mice at 20 days after surgery, were only evaluated in CORM-2 and CoPP treated animals, by using western blot assay. In these experiments sham-operated mice treated with vehicle have been used as a control.

### Drugs

CORM-2 (tricarbonyldichlororuthenium(II)dimer) was purchased from Sigma-Aldrich (St. Louis, MO), CoPP from Frontier scientific (Livchem GmbH & Co, Frankfurt, Germany) and CORM-3 (tricarbonylchloro (glycinate)ruthenium (II)) was synthesized as previously described by Motterlini et al. [21]. CORM-2 and CoPP were dissolved in dimethyl sulfoxide (DMSO; 1% solution in saline) and CORM-3 in saline. All drugs were freshly prepared before use and intraperitoneally administered in a final volume of 10 ml/kg, twice a day. The control group received the same volume of vehicle.

### Statistical Analysis

Data are expressed as mean ± standard error of the mean (SEM). For each genotype and test assessed, the comparison of the effects produced by CORM-2, CORM-3 or CoPP vs. the effects produced by vehicle in nerve-injured and sham-operated WT or NOS2-KO mice were evaluated by using a using a three way ANOVA (surgery, treatment and time as between factors of variation) followed by the corresponding one way ANOVA and the Student Newman Keuls test.

Changes in the expression of HO-1, HO-2, CD11b/c, NOS1 and NOS2 in the spinal cord and/or dorsal root ganglia from sciatic nerve-injured WT and NOS2-KO mice treated with vehicle, CORM-2 or CoPP were analyzed by using a one way ANOVA followed by the Student Newman Keuls test. A value of p<0.05 was considered as a significant.
